# 1492. Gender-Specific Targets for Primary Prevention in People Living with HIV : Applying *Life's Simple 7* to a Gender-Stratified Population of ART-Naive People Living with HIV in Tanzania

**DOI:** 10.1093/ofid/ofad500.1327

**Published:** 2023-11-27

**Authors:** Safah Khan, Gloria J Manyangu, Robert N Peck

**Affiliations:** Weill Cornell Medicine-Qatar, Maryland; Bugando Medical Centre, Mwanza, Tanzania, Mwanza, Mwanza, Tanzania; Weill Cornell Medical College, New York City, New York, USA, New York, New York

## Abstract

**Background:**

Cardiovascular disease (CVD) represents a major cause of premature mortality in people living with HIV (PLWH). However, morbidity is not equal between males and females. There is a need to better understand how gender influences cardiovascular health (CVH) in PLWH in order to identify targets for intervention. Therefore, we applied the *Life's Simple Seven* (LS7) scale to a gender-stratified cohort of ART-Naive PLWH and HIV-uninfected controls in adults.

**Methods:**

A cross-sectional analysis was conducted on a cohort of ART-naive PLWH and HIV-uninfected adults recruited from HIV clinics between June 2016 and August 2019 in Mwanza, Tanzania (HIV&HTN cohort). We applied modified *Life's Simple Seven (LS7)* definitions (**Table 1)** and compared the distribution of Ideal LS7 metrics between males and females of both study groups. Each LS7 metric was categorized as ideal (2 points), intermediate (1 point) and poor (0 point) and ordinal regressions were employed to investigate associations between gender and each individual LS7 metric. Regressions were adjusted for HIV status, age, income, education level.

**Figure:**
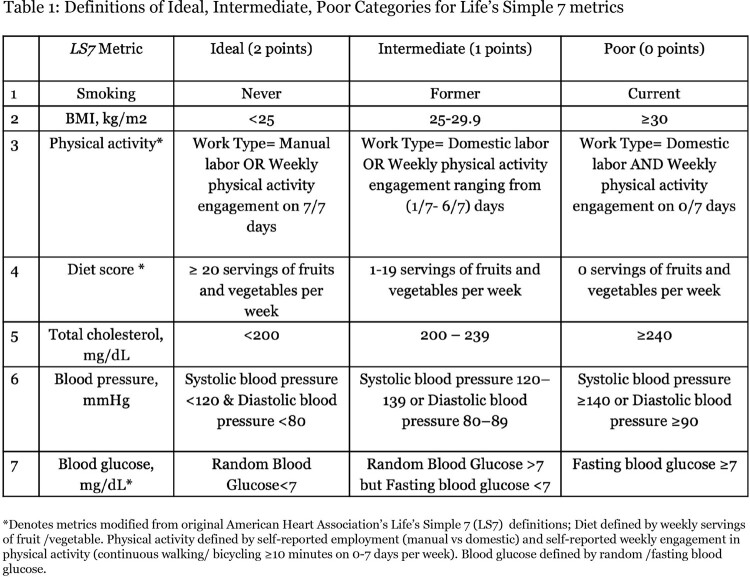

**Results:**

Our study included 997 participants, with 493 PLWH (68.7% female) and 504 HIV-uninfected (66.4% female). As displayed in **Figure 1**, **t**he distribution of Ideal LS7 metrics differed between the males and females in both study groups. Males had a higher prevalence of ideal physical activity (aOR=4.12 [95%CI=3.11 to 5.44], p< 0.001) and ideal BMI (aOR=5.10 [3.47 to 7.49], p< 0.001) but lower prevalence of ideal smoking (aOR=0.037 [0.021 to 0.065], p< 0.001) and ideal blood pressure (aOR= 0.65 [0.50 to 0.85], p=0.002). The magnitude of these differences also varied by HIV status such that they were larger in PLWH than HIV-uninfected group for smoking (41.3% vs 26.4%), physical activity (41.6% vs 24.8%) and blood pressure (12.7% vs 9.2%) metrics. By contrast, these differences were less pronounced in PLWH vs HIV-uninfected group for the BMI metric (23.1% vs 29.7%).

**Figure:**
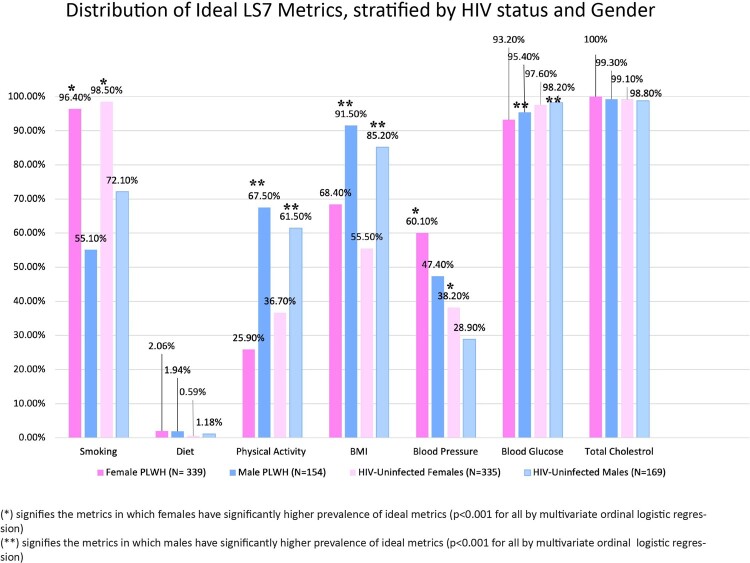

**Figure 1:** Distribution of Ideal Life's Simple 7 metrics, stratified by gender for both PLWH and HIV-uninfected controls

**Conclusion:**

Cardiovascular health profiles vary by gender and HIV status, with stark differences between male and female PLWH for most metrics. Gender-specific interventions targeting smoking cessation, blood pressure control in male PLWH and physical activity, weight loss in female PLWH are warranted to mitigate CVD risk.

**Disclosures:**

**All Authors**: No reported disclosures

